# Amino Acid Accumulation Limits the Overexpression of Proteins in *Lactococcus lactis*

**DOI:** 10.1371/journal.pone.0010317

**Published:** 2010-04-26

**Authors:** Ravi K. R. Marreddy, Eric R. Geertsma, Hjalmar P. Permentier, Joao P. C. Pinto, Jan Kok, Bert Poolman

**Affiliations:** 1 Department of Biochemistry, Groningen Biomolecular Sciences and Biotechnology Institute, Netherlands Proteomics Centre, Zernike Institute for Advanced Materials, University of Groningen, Groningen, The Netherlands; 2 Department of Molecular Genetics, Groningen Biomolecular Sciences and Biotechnology Institute, University of Groningen, Haren, The Netherlands; Baylor College of Medicine, United States of America

## Abstract

**Background:**

Understanding the biogenesis pathways for the functional expression of recombinant proteins, in particular membrane proteins and complex multidomain assemblies, is a fundamental issue in cell biology and of high importance for future progress in structural genomics. In this study, we employed a proteomic approach to understand the difference in expression levels for various multidomain membrane proteins in *L. lactis* cells grown in complex and synthetic media.

**Methodology/Principal Findings:**

The proteomic profiles of cells growing in media in which the proteins were expressed to high or low levels suggested a limitation in the availability of branched-chain amino acids, more specifically a too limited capacity to accumulate these nutrients. By supplying the cells with an alternative path for accumulation of Ile, Leu and/or Val, i.e., a medium supplement of the appropriate dipeptides, or by engineering the transport capacity for branched-chain amino acids, the expression levels could be increased several fold.

**Conclusions:**

We show that the availability of branched chain amino acids is a critical factor for the (over)expression of proteins in *L. lactis*. The forward engineering of cells for functional protein production required fine-tuning of co-expression of the branched chain amino acid transporter.

## Introduction

For several complex proteins, such as membrane proteins and multidomain proteins, the understanding of their mechanistic details is limited by the absence of a high-resolution structure. Although in part caused by difficulties in obtaining well-diffracting crystals of these proteins, for a great deal this is associated with their problematic overproduction as well. The expression of complex proteins in a functional state in well-established easy-to-use hosts like *Escherichia coli* and yeast is often a bottleneck. Although higher eukaryotic systems like insect and mammalian cells offer more opportunities for folding and post-translational modifications [1], here the production of protein in sufficient quantities for structural analysis can be a problem.

Over the past decade, the Gram-positive bacterium *Lactococcus lactis* emerged as an alternative and complementary host for heterologous protein production [2]–[7]. Lactococcal overexpression levels suffice for structure determination as demonstrated recently by the crystal structure of OppA [8]. A well-tunable nisin-inducible promoter system is available [9], which has been extensively used to produce complex proteins of both pro- and eukaryotic origin [2], [5]–[7]. As a multiple amino acid auxotroph with well-characterized transport systems for amino acids and peptides, *L. lactis* can readily be employed for incorporating amino acid analogues into proteins, as demonstrated for tryptophan analogues [10] as well as selenomethionine (SeMet) [11]. Especially the latter is of great importance if the protein produced is to be used in crystallographic studies. Next to a well characterized physiology of the expression host, one requires a chemically-defined medium (CDM) for the incorporation of amino acid analogues. Although a chemically-defined medium, supporting high growth rates, is available for *L. lactis* (Poolman and Konings, 1988), we noted a dramatic decrease in the expression levels of several proteins once the standard complex broth was changed for CDM. As this would severely limit the potential of *L. lactis* as an alternative expression platform for structural biology, we aimed at understanding and overcoming the bottlenecks limiting the functional expression of complex proteins upon growth in CDM.

To determine the limiting factor(s) in protein overexpression, we analyzed the proteomes of *L. lactis* cells grown in CDM (low expression) with those of cells grown in complex broth (high expression). We show that a too limited capacity to accumulate branched chain amino acids limits high-level expression in CDM. Reduced expression could be overcome by adjusting the composition of the CDM or rational engineering of the expression host.

## Results

### Expression levels in complex and chemically-defined growth media

**Figure 1:**
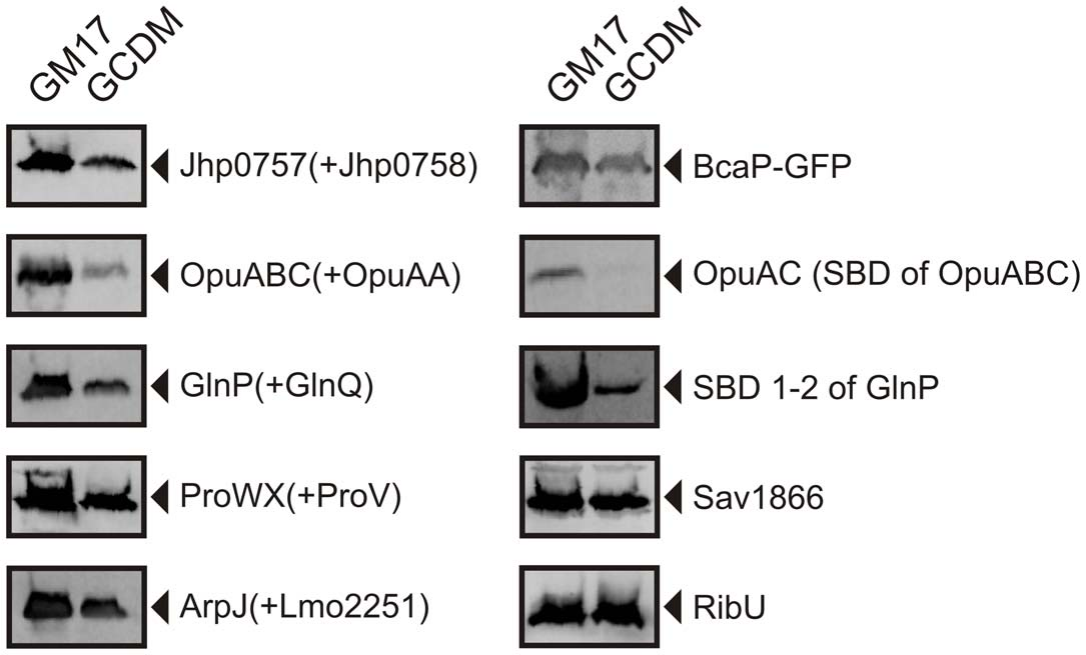
Protein overexpression in GM17- and GCDM-grown *L. lactis* NZ9000. Cells were induced at OD_600_≈0.5 with 0.1% volume of nisin A-containing NZ9700 culture supernatant and growth was continued for 2 h. Protein levels were analyzed on immunoblots, using an anti-His tag antibody. Left and right lanes in each panel represent samples from GM17- and GCDM-grown cells, respectively. For the left panels, names in brackets indicate the subunits of the respective ABC transporters that were co-expressed without a His-tag and consequently not detected on the immunoblot (SBD refers to substrate-binding domain).

Similar to *Saccharomyces cerevisiae* and *Escherichia coli* [12], [13], in *L. lactis*, we observed 3 to 10-fold diminished levels of expression levels for a significant fraction of target proteins (>50%, n>20) when a complex broth (GM17) was replaced by a rich chemically-defined medium (GCDM) [14]. Decreased levels were observed for both soluble and membrane proteins from different families (Figure 1 and Figure S1). GCDM by itself is a rich medium, like GM17, that was designed to contain ample key nutrients and support high growth rates. Consequently, the observed differences in expression levels of recombinant proteins were not anticipated. When grown in GM17 in pH-controlled bioreactors, *L.lactis* produced OpuA at ∼15 mg/L, BacP and Sav1844 at ∼4 mg/L and RibU at ∼2 mg/L, which on a mole basis corresponds to similar levels of expression. GlnPQ was produced at ∼2 mg/L, which, on a mole basis, is 7–8 fold lower than OpuA and the other proteins.

A subset of proteins, such as RibU and Sav1866, did not show the differential expression (Figure 1). Detailed analysis of the amino acid composition, hydrophobicity and codon usage of the various proteins did not provide indications that would explain why the expression of some proteins is not affected. Also, there was no correlation between the degree of differential expression and the absolute amount of protein produced.

### Screening for improved expression

**Figure 2:**
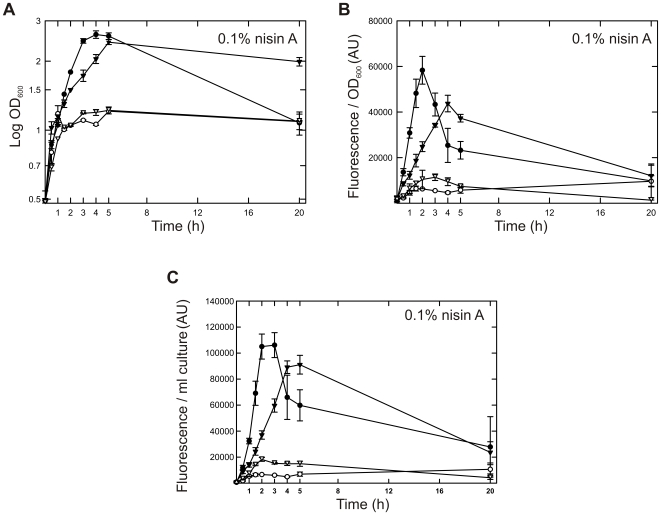
Time-resolved protein expression in GM17- and GCDM-grown cells. (**A**) Growth of *L. lactis* NZ9000 in GM17 (closed symbols) and GCDM (open symbols), following the addition of 0.1% of nisin A-containing NZ9700 medium supernatant to a culture at OD_600_≈0.5. The cells express either OpuAC-GFP (circles) or BcaP-GFP (inverted triangles). Growth of the cells was monitored by measuring the optical density at 600 nm. (**B and C**) Time dependence of OpuAC-GFP and BcaP-GFP expression (symbols the same as in panel A). Expression levels were quantitated on the basis of GFP fluorescence and normalized on the basis of cell density (**panel B**) or culture volume (**panel C**).

To improve the production of the proteins whose expression was decreased in GCDM-grown cells, we initially determined how expression was affected by inducer concentration and induction time. Figure 2 and Figure S2 show representative data for the expression of the soluble substrate receptor OpuAC and the membrane transporter BcaP in both GM17- and GCDM-grown cells. The proteins were produced as a GFP fusion protein, which facilitated the screening and allowed the quantification of functionally expressed protein [15], [16]. The C-terminal GFP moiety did not affect the expression of the proteins nor their activity. Maximal expression, corrected for differences in cell density, was generally observed between 2–4 hours of induction (Figure 2B; Figure S2B and S2D). Clearly, the decreased expression levels in GCDM were not restored under any condition; expression was highest in GM17 although in this complex broth the expression level peaked more prominently than was observed in GCDM. Alternative approaches to improve the expression levels in GCDM, such as increasing the concentrations of individual or combinations of amino acids, vitamins, precursors of nucleotides and/or minerals failed as well (data not shown). We thus needed a more comprehensive approach to identify the bottlenecks for high level expression in GCDM.

### Comparison of the soluble proteome of GM17 and GCDM grown cells

To understand the underlying basis for the differences in expression levels in GM17- and GCDM-grown cells, we compared the soluble proteomes of *L. lactis* cells grown in these two media. Cells were grown in GCDM and GM17 in biological triplicates in a pH-controlled bioreactor. The growth rates of the cells (*μ_max_*) were 1.21±0.08 and 0.86±0.03 hr^−1^ for GM17 and GCDM, respectively. The soluble proteomes of cells in mid-exponential phase (OD_600_≈0.5) were analyzed using the 2D-DIGE methodology. Proteins were separated in the first dimension by iso-electric focussing in the pH range of 4–7 and 7–11 followed by 15% SDS-PA gel electrophoresis as the second dimension. As membrane proteins are not resolved by 2D gel electrophoresis and some proteins fall outside the pH range used, this procedure would in theory allow us to resolve 63% of the lactococcal proteome.

**Figure 3:**
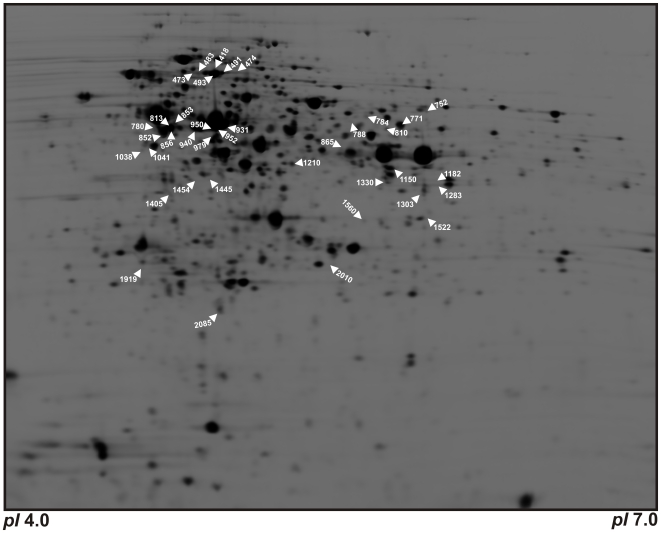
Reference soluble proteome map of *L. lactis* NZ9000 grown in GM17 and GCDM medium. The pI increases from 4.0 on the left to 7.0 on the right. High molecular weight proteins are in the upper part of the figure, low molecular weight proteins in the lower region. Differentially expressed proteins are annotated by spot numbers; their protein description is given in Table S1.

Analysis of the gels in the pH range of 4–7 showed for 54 spots a significant differential abundance (t-value <0.01) with an average ratio greater than 1.5-fold up or down. In the pH range of 7–11 no significant differences in spot volumes were observed. Using MALDI-TOF/TOF MS, we could assign proteins to 39 of the 54 affected spots (indicated in Figure 3 and Table S1). In total, 24 unique proteins were identified out of which 19 were up-regulated and 5 were down-regulated upon growth in GCDM relative to GM17.

A small fraction of this set of differentially expressed proteins is involved in glycolysis. Pgk, phospho-glycerate kinase, and FbaA, fructose-biphosphate aldolase, were upregulated upon growth in GCDM, whereas expression of GalU, UDP-glucose-phosphate uridilyltransferase, and FruC, fructose-phosphate kinase were downregulated. Despite these changes, the rate of glycolysis, the type of glycolytic end-product (>95% lactate) and the magnitude of the membrane potential did not differ between GCDM- and GM17-grown cells (Figure S3), suggesting that carbon metabolism and energy status were not affected to a great extent by the different media.

The majority of the differentially-expressed proteins were involved in various stages of peptide and amino acid metabolism (Table S1). The proteins that were upregulated upon growth in GCDM include cytosolic peptidases (PepO, PepC, PepQ); enzymes involved in biosynthesis of amino acids (LysA, IlvE, IlvC, AraT, and GltD); GatA, an amino acyl tRNA synthetase; and proteins involved in transcription (GreA, a transcriptional elongation factor) and translation (TufA, an elongation factor, and the ribosomal proteins L10 and S1). Of this set, the expression of PepO, PepC, LysA, IlvC, AraT and GltD, is controlled by CodY, a global regulator of nitrogen metabolism in *L. lactis* and other Gram-positive bacteria [17]. The aminopeptidase PepN, also regulated by CodY in wildtype *L. lactis*, is not present in the strain (NZ9000) used here, as this locus was used for the chromosomal integration of the *nisRK* genes [18], *i.e.* the two-component regulatory system required for nisin A induction. The only proteins downregulated in GCDM are involved in arginine and proline metabolism (arcB and proC, respectively).

### Casitone restores the overexpression levels in GCDM-grown cells

**Figure 4:**
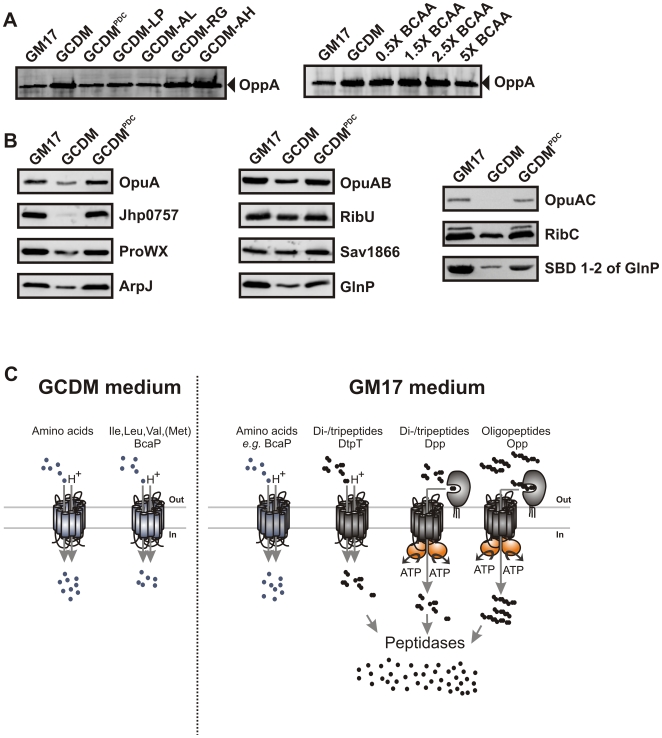
Casitone restores the overexpression levels in GCDM-grown cells. (**A**) Analysis of OppA levels in total cell lysates from *L. lactis* NZ9000; immunoblots were developed using an anti-OppA antibody. Cells were grown in GM17, GCDM, GCDM^PDC^ or GCDM with various dipeptides (left panel; amino acids indicated by their one letter code) or GCDM with altered branched-chain amino acids concentrations (1X BCAA is the standard BCAA concentration in GCDM). (**B**) Effect of supplementation of GCDM with casein hydrolysate (GCDM^PDC^; right lanes) on (membrane) protein expression in *L. lactis* NZ9000; GM17- (left lanes) and GCDM-grown cells (middle lanes) are shown for comparison. Cells were induced at OD_600_≈0.5 with 0.1% volume of nisin A-containing NZ9700 culture supernatant and growth was continued for 2 h. Protein levels were analyzed on immunoblots, using an anti-His tag antibody. For the left panels, names in brackets indicate the subunits of the respective ABC transporters that were co-expressed without a His-tag and consequently not detected on the immunoblot. Sav1866 and RibU are examples of proteins whose expression is not affected by the medium. (**C**) Schematic representation of the uptake pathways for amino acids and peptides in *L. lactis*.

The comparison of the proteomes of GCDM- and GM17-grown cells suggested a critical role for CodY as many differentially expressed proteins are part of the *codY* regulon. As the repressing activity of CodY is known to be relieved by low intracellular concentrations of branched chain amino acids (BCAAs) [17], [19], the proteomic data would be consistent with a limitation in availability of leucine, isoleucine and/or valine upon growth in GCDM. To analyze how medium composition affects the activity of CodY, we used the oligopeptide-binding protein OppA as a facile marker for the (de)repressing activity of CodY. The transcriptional regulation of the *opp-pep* operon harboring *oppA* is tightly controlled by CodY [19]. Indeed, compared to GM17, expression levels of OppA were approximately 5-fold increased upon growth in GCDM (Figure 4A, left panel).

To test whether the potential limitation of nitrogen in GCDM could be relieved, we initially supplemented the medium with casitone, a proteolytic hydrolysate of casein. OppA levels were significantly reduced upon addition of pancreatic digest of casein to GCDM (GCDM^PDC^) and similar to those in GM17-grown cells (Figure 4A, left panel). Apparently, the addition of casitone to GCDM leads to an increase in the intracellular concentration of BCAAs and consequently an increase in the repressing activity of CodY. As BCAAs are present in millimolar concentrations in GCDM and only a fraction is used for growth, these results point to a limited capacity of *L. lactis* to take up BCAAs from the medium.

Next, we compared the recombinant protein production levels in GM17, GCDM and GCDM^PDC^-grown cells. For all proteins tested, the reduced expression levels in GCDM were restored to high levels in GCDM^PDC^ (Figure 4B). Taken together, these results suggest that a low intracellular concentration of BCAAs or the resulting decrease in the repressing activity of CodY cause the decreased protein overexpression levels in GCDM.

### BCAA-containing dipeptides restore the overexpression levels in GCDM-grown cells

Since GCDM^PDC^ has a complex nitrogen source (amino acids and peptides), further attempts were made to simplify the medium. Increasing (or decreasing) the levels of BCAAs in GCDM up to 2.5-fold did not affect the OppA levels (Figure 4A). Only a five-fold increased BCAA concentration led to a slight decrease in OppA expression, but the expression levels of recombinant proteins were not increased when *L. lactis* was grown in this medium (data not shown).

To allow cells to accumulate BCAAs via alternative mechanisms (Figure 4C), that is, not only via a BCAA carrier that is already saturated at submillimolar concentrations of the corresponding amino acids [14], we supplemented GCDM with several dipeptides. Inside the cell, peptides are rapidly hydrolyzed to amino acids, and transport of BCAA-containing peptides is thus expected to yield increased intracellular levels of Leu, Ile and Val, the known co-repressors of CodY. OppA levels were low in GCDM supplemented with Leu-Pro or Ala-Leu, but high in the presence of Arg-Gly or Ala-His (Figure 4A), indicating that BCAA-containing dipeptides increased the CodY repression activity.

**Figure 5:**
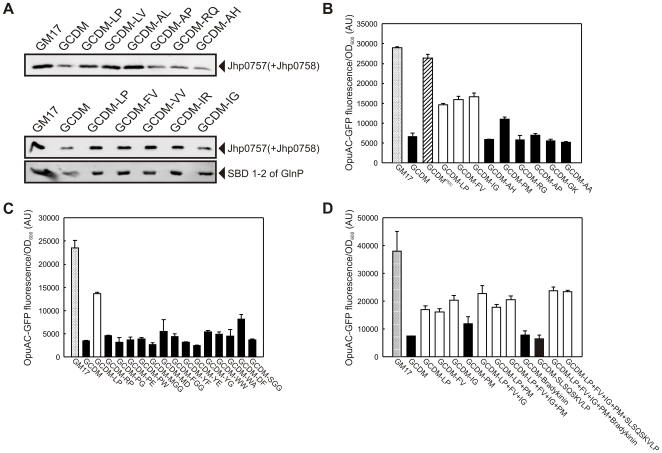
Peptides with BCAAs increase the recombinant protein production in GCDM grown cells. (**A**) Effect of supplementation of GCDM with dipeptides on the expression levels of the ABC transporter Jhp0757 and the SBDs of the ABC transporter GlnPQ (SBD1-2 of GlnP) in *L. lactis* NZ9000 cells. Cells were induced at OD_600_≈0.5 with 0.1% volume of nisin A-containing NZ9700 culture supernatant and growth was continued for 2 h. Protein levels were analyzed on immunoblots, using an anti-His tag antibody. Growth media used are indicated above the lanes. (**B, C and D**) Effect of peptides on the expression level of the soluble protein OpuAC-GFP in *L. lactis* NZ9000 grown in GM17, GCDM and GCDM^PDC^ or GCDM supplemented with peptides. GCDM supplemented with di-/tripepides containing at least one branched-chain amino acid are indicated by white bars. Whole cell GFP fluorescence of *L. lactis* NZ9000 carrying pNZ(OpuAC-GFP) was used to quantitate OpuAC-GFP expression levels. Amino acids are indicated by their one-letter code.

Next, we tested the effect of dipeptides on the production of recombinant protein. Of all additives tested, an increase in the protein overexpression levels was only observed in GCDM supplemented with dipeptides containing at least one BCAA (Leu-Pro, Leu-Val, Ala-Leu, Phe-Val, Val-Val, Ile-Arg, and Ile-Gly). Dipeptides devoid of BCAAs had no effect (Figure 5 and Figure S4). Importantly, a combination of two or more dipeptides containing different BCAAs did not enhance the expression levels much further than single peptide(s) containing one BCAA (Figure 5D). Furthermore, addition of the casein-derived, BCAA-containing oligopeptide SLSQSKVELP or the BCAA-free oligopeptide bradykinin (RPPGFSPFR) did not significantly affect the expression levels (Figure 5D). The latter most likely results from the relatively low oligopeptide transport rates, which are at least an order of magnitude lower than the uptake rates for di/tripeptides [20], [21].

### Overexpression of a BCAA transporter enhances protein overproduction

**Figure 6:**
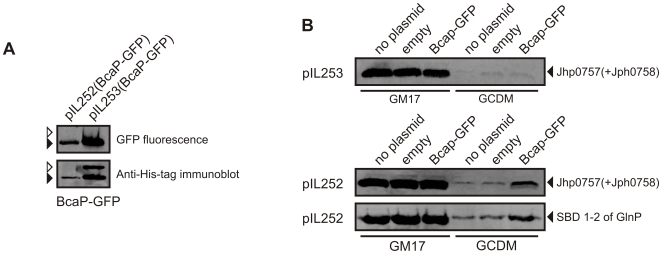
Co-overexpression of BCAA transporter enhances the levels of recombinant protein production in GCDM grown cells. (**A**) High expression of BcaP-GFP using the high copy vector pIL253 exceeds the capacity of the cell to correctly fold the protein. Expression of the gene coding for BcaP-GFP was controlled by the constitutive *P_32_* promoter in a low (pIL252) or high (pIL253) copy vector. Whole cells samples of *L. lactis* NZ9000 containing either plasmid were analyzed by in gel GFP fluorescence, to detect the well-folded BcaP-GFP (open arrow), and immunoblotting, to detect both the folded and unfolded (closed arrow) species. (**B**) Co-expression of BcaP-GFP using the pIL252 vector enhances recombinant protein production. Expression levels of the proteins that are indicated on the right of the panels, were analyzed in *L. lactis* NZ9000 harboring no secondary plasmid (no plasmid), an empty secondary plasmid (empty), or a pIL vector expressing *bcaP-GFP* from the constitutive *P_32_* promoter. Growth media and vector types are indicated on the bottom and the left of each panel, respectively. For cells with pIL252/253 vectors the medium was supplemented with 5 µg/ml of erythromycin in addition to 5 µg/ml of chloramphenicol (for maintenance of the pNZ-derived vectors). Expression levels were determined by immunoblotting using an anti-His antibody.

If, in GCDM-grown cells, the cytosolic pools of BCAAs determine the synthesis of heterologous proteins, then the limitation should be relieved by increasing the capacity to take up BCAAs from the medium. Of the two transporters for BCAAs in *L. lactis*, BcaP and BrnQ, the fast majority of Ile, Leu and Val is taken up via BcaP [22]. We overexpressed BcaP by placing the gene coding for BcaP-GFP into a low (pIL252) and high (pIL253) copy number vector under the control of the constitutive *P_32_* promoter. Expression of BcaP-GFP was highest using the high copy pIL253 vector and even exceeded the capacity of the cell to correctly fold the protein, as based on the presence of a non-fluorescent BcaP-GFP species [23] (Figure 6A). Expression of BcaP-GFP from pIL252 allowed only low production levels of the transporter and a modest increase of the BCAA transport capacity compared to the empty plasmid control (data not shown). While co-expression of BcaP-GFP from the high-copy pIL253 did not increase the expression levels of our target protein, co-expression using the low-copy pIL252 vector did increased the protein overexpression levels in GCDM-grown cells, although not completely (Figure 6B). While these data emphasize the limited transport capacity for BCAAs in wildtype *L. lactis* and its consequences for protein overexpression, they also show that fine-tuning the co-expression of critical factors such as BcaP is an essential factor in the forward engineering of cells for functional protein production.

### Derepression activity of CodY affects the recombinant protein expression

**Figure 7:**
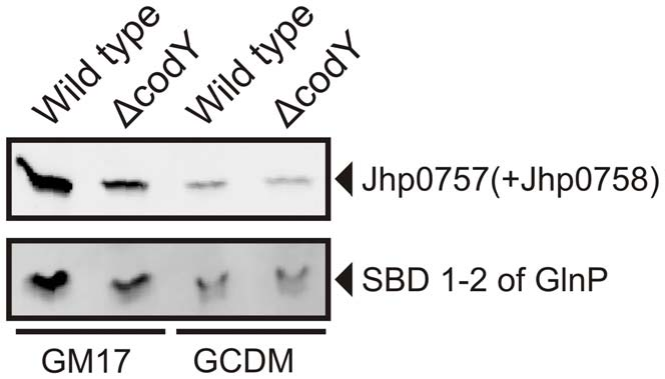
Derepression activity of CodY influences the recombinant protein production. Overexpression of recombinant proteins in *L. lactis* NZ9000 and NZ9000Δ*s* cells grown in GM17 and GCDM. Cells were induced at OD_600_≈0.5 with 0.1% volume of nisin A-containing NZ9700 culture supernatant and growth was continued for 2 h. Protein levels were analyzed on immunoblots, using an anti-His tag antibody.

Thus far, the evidence presented for the role of CodY in decreasing the heterologous protein production levels was indirect. To circumstantiate the importance of CodY, we constructed the *codY* knock-out strain NZ9000Δ*codY* and analyzed the protein levels in this host. The protein overexpression levels in NZ9000Δ*codY* were significantly reduced compared to those in the isogenic NZ9000 upon growth in GM17, whereas similar levels were observed in GCDM (Figure 7). These results establish a direct relationship between the activity of CodY and the overexpression levels of heterologous proteins. A decrease of the repressing activity of CodY, as observed upon growth in GCDM or upon deletion of the entire *codY* gene, results in lower overexpression levels of recombinant proteins. Notably, the expression levels in NZ9000*ΔcodY* grown on GM17 were not as low as observed for NZ9000 and NZ9000*ΔcodY* in GCDM (Figure 7).

## Discussion

A versatile mature protein overexpression system requires the availability of growth media that support fast growth and high protein production levels. A thorough understanding of the factors that determine expression levels and strategies to overcome bottlenecks are important for predictable protein production. Next to complex media, a defined growth medium is indispensable for manipulations of the protein produced. For instance, to fully exploit the potential of a host for production of proteins to be used in crystallographic studies (*i.e.*, experimental phasing), one needs to be able to incorporate selenomethionine (SeMet) into expressed proteins [11]. Similarly, the incorporation into proteins of the wide variety of amino acid analogs available nowadays [24] is critically dependent on chemically-defined growth medium. In this paper, we report the cause for reduced overexpression levels of heterologous proteins by *L. lactis* in a chemically-defined medium and show that the uptake capacity of the organism for branched-chain amino acids limits protein synthesis. Strong indications for a limitation of the overexpression levels by a low intracellular concentration of BCAAs came from a proteomic analysis of cells grown in GCDM and GM17, which revealed an upregulation in GCDM of several proteins part of the CodY-regulon. Proof for the hypothesis that BCAA accumulation limits protein synthesis came from physiological and engineering studies. Importantly, by supplementing the medium with BCAA-containing dipeptides or the engineering of an increased uptake capacity for Ile, Leu and Val we could elevate the expression levels of heterologous proteins.

The proteomic analysis showed that the expression levels of PepO, PepC, LysA, AraT, GltD, IlvC, and OppA are significantly higher in cells grown in GCDM than in GM17 (Table S1 and Figure 4A), consistent with an adaptive mechanism in which the cell scavenges and utilizes amino acids as efficiently as possible. The transcription of these genes is controlled by the activity of the pleiotropic regulatory protein CodY, which is effected by the intracellular concentrations of BCAAs [19]. Guided by the proteomics data, experiments were designed to overcome the apparent limitation in amino acid accumulation capacity in GCDM-grown cells. GM17 contains polypeptone, phytone soy peptone, yeast- and beef-extract as nitrogen source, whereas GCDM contains free amino acids [14], [25]. Addition of casitone, a complex mixture of peptides, to the GCDM (yielding GCDM^PDC^) reduced the expression levels of CodY targets such as OppA and increased the expression of recombinant proteins up to the levels observed in GM17. Although this observation confirmed that a decreased CodY response, that is a higher repression of transcription of CodY-regulated genes, improves protein overexpression, this strategy would compromise the chemically-defined nature of GCDM.

As affinity constants for amino acid transport in *L. lactis* are in the low micromolar range [26], transport would be expected to proceed at maximum rates at the millimolar concentrations present in GCDM. However, Ile, Leu, Val and Met all compete with each other for the same transport system and the individual fluxes will not only be determined by the affinity constants but also by the relative amounts of the amino acids in the medium [14]. Thus, increasing or decreasing the BCAA concentration is not readily expected to affect the CodY activity. Supplementing GCDM with dipeptides provides an additional path for BCAA accumulation and did decrease the CodY response, but expression levels were not fully restored to that of GM17 grown cells. Presumably GCDM^PDC^ offers a more optimal nitrogen source than GCDM supplemented with one or a few BCAA-containing dipeptides. As in GCDM no strong additive effect was observed upon the addition of a second dipeptide containing a different BCAA, the decreased expression levels seem to be at least partly resulting from a CodY-mediated regulatory mechanism, rather than only a direct limitation of the protein synthesis rate by the low intracellular BCAA concentration.

Next to adjustments of the growth medium, modifications of the expression host were explored. A moderate increase in the level of the BCAA transporter BcaP did increase protein overexpression, while higher BcaP expression levels did not show any effect on the heterologous expression levels. While higher levels of BcaP resulted in higher uptake rates of BCAAs, this positive effect was probably compensated for by a larger negative effect caused by the high overproduction of BcaP-GFP which apparently became a burden to the cell as part of the protein got misfolded (Figure 6A; [27].

Lactic acid bacteria are multiple amino acid auxotrophs; Ile, Leu, Val, Met, Gln/Glu and His are essential for *L. lactis* but at least another four amino acids are needed for high rates of growth [14]. In GCDM-grown cells, most of the protein is synthesized from exogenous amino acids. With amino acid concentrations in the millimolar range and K_M_ values for Ile, Leu, Val and Met of 8, 6.5, 12 and 113 µM, respectively, [26], the concentrations in GCDM would not seem limiting per se. However, for a cell to grow with a rate of 1.0 h^−1^ (doubling time of 41 min), the rate of BCAA transport (V*_t_*) should be at least 40–50 nmol/min×mg of protein as these amino acids and Met compete for the same transport system [14]. This calculated rate is close to what is experimentally observed for glycolyzing cells of *L. lactis* NZ9000 [22], and thus suggests that in GCDM CodY-mediated regulation and/or protein synthesis are controlled (limited) by the intracellular BCAA concentrations.

Why are BCAA-containing dipeptides so effective in reducing the CodY response and stimulating heterologous protein production? In *L. lactis*, BCAAs are primarily taken up by the BcaP protein, a proton motive force-driven transporter [22], [26]. For uptake of peptides, *L. lactis* uses the proton motive force-driven DtpT system (di- and tripeptides; [28]) and the ABC transporters Dpp (di- and tripeptides) and Opp (oligopeptides; [20]). Internalized peptides are cleaved to individual amino acids by intracellular peptidases, which are then available for protein synthesis. Of these transport systems, DtpT has an activity that is at least an order of magnitude higher than that of the others [20], [21] and thus contributes immensely to the accumulation of BCAAs when present in di- or tripeptides.

### In conclusion

By combining proteomic and physiological approaches we identified a major bottleneck in the path for synthesis of functional proteins in *L. lactis*. By rational modification of the growth medium or forward engineering of the cell towards branched-chain amino acid uptake, we could increase the capacity to synthesize recombinant proteins. A mere increase in the intracellular BCAA concentration, provided via dipeptide uptake, sufficed in reducing the CodY response and increased the production of recombinant (membrane) proteins. Moreover, deletion of the *codY* gene in *L. lactis* significantly reduced protein expression levels in GM17-grown cells. In these cells, the CodY-mediated repression mechanism is no longer operative, which may lead to uncontrolled synthesis of enzymes involved in nitrogen metabolism and place a burden on any recombinant protein production.

## Materials and Methods

### Bacterial strains and growth conditions

*Lactococcus lactis* strain NZ9000 and derivatives were grown at 30°C in M17 (Difco, Detroit, MI, USA) or chemically-defined medium (CDM) [14] supplemented with 1% glucose (w/v), hereafter referred to as GM17 and GCDM, respectively. When specified, GCDM was supplemented with either 1.5% (w/v) pancreatic digest of casein (GCDM^PDC^; Difco, Detroit, MI, USA), 1 mM of specified di-peptides, or 100 µM of the oligopeptides RPPGFSPFR (bradykinin) or SLSQSKVELP (Bachem AG, Bubendorf, Switzerland). Growth media were supplemented with 5 µg/ml chloramphenicol or 5 µg/ml erythromycin when appropriate. *Escherichia coli* DH5α strain, used as intermediary cloning host, was grown at 37°C under vigorous aeration in Luria broth supplemented with 150 µg/ml erythromycin.

### Plasmid construction

DNA manipulations were done according to standard procedures. Plasmid and primer sequences used in this study are specified in Table 1 and Table S2, respectively. Ligation-independent cloning in pREcLIC and pREcLIC-GFP and the subsequent conversion of these plasmids into lactococcal expression vectors, using the Vector Backbone Exchange (VBEx) procedure, was performed as described [2]. Plasmid pREcLIC-GFP(P_32_-*bcaP*) was prepared by substituting the *Bgl*II-*Nco*I fragment containing the *nisin A* promoter in pREcLIC-GFP(*bcaP*) with the *P_32_* promoter, which was obtained by PCR amplification using pAMP31 as template [29]. The sequence containing the *P_32_* promoter plus *bcaP-GFP* from pREcLIC-GFP(*P_32_*-*bcaP*) was PCR amplified with flanking *Xho*I and *Eco*RI sites, digested and ligated with the *Xho*I – *Eco*RI fragments of pIL252 or pIL253 [30], resulting in the formation of pIL252(*P_32_-bcaP-gfp*) and pIL253(*P_32_-bcaP-gfp*).

**Table 1:** Summary of the plasmids used in this study.

Plasmid	Gene	Function of protein	Organism	Reference
pNZ(*opuA*His)	*opuA*	Transport of glycine betaine	*Lactococcus lactis*	[37]
pNZ(*opuAC*His)	*opuAC*	SBD of OpuA	*Lactococcus lactis*	[38]
pNZ(*opuAC-GFP*His)	*opuAC-GFP*	SBD of OpuA fused to GFP	*Lactococcus lactis*	This work
pNZ(*ribU*His)	*ribU*	Transport of riboflavin	*Lactococcus lactis*	[39]
pNZ(*ribC*His)	*ribC*	Conversion of riboflavin to cofactor (FMN and FAD)	*Lactococcus lactis*	Laboratory stock
pNZ(*SBD1&2*His*glnP*)	*glnP*(*SBD1&2*)	SBDs of glutamine transporter GlnPQ	*Lactococcus lactis*	Laboratory stock
pREcLIC(*jhp0757*His)	*jhp0757*	Putative osmoregulatory transporter	*Helicobacter pylori*	This work
pREcLIC(*proWX*His)	*proWX*	Transport of choline	*Streptococcus pneumoniae*	This work
pREcLIC(*arpJ*His)	*arpJ*	Function unknown	*Listeria monocytogenes*	This work
pREcLIC(*glnP*His)	*glnP*	Transport of glutamine	*Lactococcus lactis*	This work
pREcLIC(*sav1866*His)	*sav1866*	Multidrug transporter	*Staphylococcus aureus*	Laboratory stock
pREcLIC(*bcaP-gfp*His)	*bcaP-gfp*	Transport of branched chain amino acids	*Lactococcus lactis*	Laboratory stock
pIL252(*P_32_*-*bcaP-gfp*) & pIL253(*P_32_*-*bcaP-gfp*)	*bcaP-gfp*	Transport of branched chain amino acids	*Lactococcus lactis*	This work
pIL252(*P_32_*-*bcaP-gfp*His) & pIL253(*P_32_*-*bcaP-gfp*His)	*bcaP-gfp*	Transport of branched chain amino acids	*Lactococcus lactis*	This work

*NBD, Nucleotide-binding domain; TMD, Transmembrane domain; SBD, Substrate-binding domain.

pNZ-derived plasmids are based on pNZ8048 [18], pREcLIC-derived plasmids are based on [2] and pIL-derived plasmids are based on [30]. His, indicates the hexa histidine tag engineered at the C-terminal of the protein.

### Strain manipulation

For deletion of the *codY* gene in *L. lactis* NZ9000, we used the procedure described by [31]. DNA fragments flanking the *codY* gene, that are, the ∼700 bp *Xba*I-*Cla*I upstream and the ∼1000 bp *Cla*I-*Kpn*I downstream fragments, were obtained by PCR amplification from genomic DNA. The PCR products were cut with the appropriate restriction enzymes and ligated into plasmid pCS1966 [31]. The resulting plasmid pCS1966Δ*codY* was introduced in *L. lactis* NZ9000. Cells in which the plasmid was integrated into the chromosome were selected on GM17 plates with erythromycin. Positive colonies were grown in synthetic medium (SA medium) [32] and plated on SA agar plates with 10 µg/ml of 5-fluoro-orotate, thereby selecting for *L. lactis* NZ9000Δ*codY* strains in which the *codY* gene is deleted. The deletion of the *codY* gene was confirmed by PCR and phenotypic analysis.

### Protein expression, immunodetection and whole cell fluorescence

For overexpression, cells were grown until OD_600_≈0.5 and induced with 0.001 volume of the nisin A-containing, filtered culture supernatant of *L. lactis* NZ9700 and allowed to grow for 2 h before harvesting. Sample volumes were adjusted so that ∼1 mg whole cell protein was taken for all samples. Cells were washed once with 100 mM KPi, pH 7.0, and resuspended in 1 ml of ice-cold 100 mM KPi, pH 7.0, 10% (v/v) glycerol, 1 mM MgSO_4_, 1 mM PMSF and trace amounts of DNAseI. After the addition of 300 mg of glass beads (∼100 µm diameter), cells were lysed by three rounds of bead beating in a Fastprep machine at force 6.5 for 30 s with cooling intervals of 5 min on ice in between. Four volumes of cell lysate were mixed with one volume of 5X SDS-PAGE loading buffer. Protein samples were resolved on 12.5% SDS-PAGE gels, and in-gel GFP fluorescence was determined with a LAS-3000 imaging system (Fujifilm, Minato-ku, Tokyo, Japan). His-tagged proteins were detected by immunodetection with a primary monoclonal antibody (1∶5000) raised against the hexa His-tag (GE Healthcare, Uppsala, Sweden). OppA expression levels were detected with a polyclonal antibody (1∶10000) raised against OppA. Chemiluminescence detection was done using the Western-light kit with CSPD (Tropix Inc, Bedford, MA, USA) as substrate and imaging with the Fujifilm LAS-3000 imaging system.

For measurements of whole cell GFP fluorescence, nisin A-induced cells were harvested 2 h after induction, washed twice with 100 mM KPi, pH 7.0, and resuspended in 100 mM KPi, pH 7.0 to a final OD_600_ of 1. Whole cell fluorescence was determined on a 200 µl sample in an FL600 microplate fluorescence reader (Bio-Tek instrument, Winooski, VT, USA) with an excitation wavelength and emission filter of 485 nm and 530/25 nm, respectively.

### Proteomics analyses

To assure a homogeneous culture, each initial pre-culture (5 ml) on GM17 and GCDM was inoculated with a single colony obtained from a streak of a frozen stock of *L. lactis* NZ9000/pNZ8048 on a GM17 agar plate. A 1% inoculum of the initial pre-culture (grown for 8–10 h) was transferred to 50 ml of the respective media (1∶100 dilution) and subsequently serial dilutions of 1∶1000 and 1∶10000 were prepared and incubated overnight. The next day, cell densities were determined by measuring the OD_600_, and a 1% inoculum of cultures in early log phase (OD_600_ = 0.2–0.4) was used to inoculate 2 l of the respective medium in bioreactors (Applikon, Schiedam, The Netherlands). The culture was stirred at 400 rpm and the pH was automatically maintained at 6.8 by the addition of 4 M KOH. One liter of culture of cells in the mid-exponential (OD_600_ = 0.5) phase was collected, supplemented with chloramphenicol (100 µg/ml final concentration) to prevent protein synthesis and harvested by centrifugation at 8281×*g* for 15 min at 4°C. The pellet was washed once with ice-cold 50 mM KPi, pH 7.0, and resuspended in 5 ml of the same buffer. Cells were lysed by three passes through a pre-cooled small French Press cell (Thermo IEC, Waltham, MA, USA) at 12,500 psi. Whole cells were removed by centrifugation at 7,650×*g* for 15 min and membrane fragments were removed by centrifugation at 267000×*g* for 1 hr. The supernatant containing the soluble proteome was aliquoted (500 µl volumes), frozen in liquid nitrogen and stored at −80°C.

### 2D gel electrophoresis

The protein concentration of the samples was determined using the 2D-quant kit (GE Healthcare, Uppsala, Sweden). Oligonucleotides were degraded by incubating a volume equivalent to 1 mg of protein with 1 µg DNAse I and 1 µg RNAse I at room temperature for 1 hr. Subsequently, proteins were precipitated by the addition of four volumes of ice-cold acetone and overnight incubation at −20°C. Precipitation of proteins was performed to remove salts from the sample, which affect the iso-electric focusing step. The protein recovery after acetone precipitation was ∼80%. Precipitated proteins were collected by centrifugation at 16100×*g* for 20 min at 4°C and resuspended in approximately 80 µl of 7 M urea, 2 M thiourea, 4% (w/v) CHAPS and 30 mM Tris-HCl, pH 8.5 (Labeling Buffer). The pH of the sample was adjusted to pH 8.5 using Labeling Buffer, pH 9.5, and the final volume was adjusted to a protein concentration of 5 mg/ml with labeling buffer, pH 8.5. For labeling, 50 µg protein of each sample was incubated with 400 pmol CyDye (dissolved in dimethylformamide, GE Healthcare, Uppsala, Sweden), at 4°C in the dark for 30 minutes and subsequently the reaction was quenched by the addition of 1 mM L-lysine (final concentration). A bias in the final results due to specific properties of the fluorophores was avoided by a dye-swap: part of the samples derived from the cultivations in GM17 and GCDM were labeled with the Cy3-dye, while for the other part the Cy5-dye was used. An internal standard was prepared by pooling an equal amount of protein from all 6 samples and labeling these with the Cy2-dye as described above. For electro-focusing, 40 µg of Cye2-, Cye3- and Cye5-labelled samples of each set (internal standard, GM17, GCDM) were mixed together and loaded on IPG strips with pH range 4–7 (GE Healthcare, Uppsala, Sweden) by the overnight rehydration method [33]. For preparative gels, 400 µg of unlabelled protein was spiked with 50 µg of Cy2-labelled protein and was loaded on the IPG strips by the cup loading method [33]. Strips were focused in an Ettan IPGphor (GE Healthcare, Uppsala, Sweden) with a 0–1000 V gradient for 3 h, stabilization at 1000 V for 1 hr, a gradual increase from 1000 to 8000 V for 30 min and final focusing at 8000 V for 6 h (48000 Vhrs). Focused strips were equilibrated with 6 M urea, 50 mM Tris-HCl, pH 8.8, 30% (w/v) glycerol and 2% SDS (w/v) in two steps with initial reduction of cysteine disulfide bridges with 10 mg/ml DTT, followed by alkylation with 25 mg/ml iodoacetamide. Second dimension SDS-PAGE was performed in an Ettan DALTtwelve (GE Healthcare, Uppsala, Sweden) on 15% acrylamide gels cast on low fluorescence glass plates (GE Healthcare, Uppsala, Sweden) coated with bind silane.

### Protein visualization and image analysis

CyDye labeled proteins were visualized in-gel using a Typhoon 9400 scanner (GE Healthcare, Uppsala, Sweden) with excitation wavelength and emission filter of 488 nm and 520 nm with band pass 40 nm for Cye2, 532 nm and 580 nm with band pass 30 nm for Cye3 and 633 nm and 670 nm band pass 30 nm for Cye5. Images were cropped to the area of interest showing a consistent spot pattern, and speckles were removed by filtering in Image quant software (GE Healthcare, Uppsala, Sweden). Images were analyzed using Decyder Differential Analysis Software, version 6.5 (GE Healthcare, Uppsala, Sweden) to compare prominent changes in the proteome within the triplicate samples of cells grown in GM17 and GCDM. Detection of gel spots, matching of spots between gels and determination of the relative abundance of a spot within a gel was performed using the Differential In gel Analysis (DIA) module. The DIA module determines the relative abundance of a spot within a gel set by comparing the ratio of the Cy3 and Cy5 signal within a gel and normalizing this to the Cy2 signal. The results of the analyses of the gels of the biological triplicates analyzed with the DIA module were collected in the Biological Variation Analysis (BVA) module where the statistical analysis was performed. Protein spots with an average intensity ratio greater than 1.5 and with a t-value <0.01 were selected as spots of interest.

### Protein identification by mass spectrometry

For identification, protein spots of interest were picked into a 96 well plate using the Ettan spot picker (GE Healthcare, Uppsala, Sweden) equipped with a 2 mm diameter picker head. Gel plugs were washed with 50 mM ammonium bicarbonate, pH∼7.6, followed by dehydration with acetonitrile and drying in a vacuum centrifuge. The dried gel plugs were allowed to re-swell at 4°C for 30 min after the addition of 2.5 µl of 12.5 ng/µl trypsin solution (Sequencing grade modified porcine trypsin, Promega, Madison, WI, USA). Next, 5 µl of 25 mM ammonium-bicarbonate pH∼7.6 was added and the gel plug was incubated at 37°C. After overnight incubation, the sample was sonicated in a water bath for 10 min and 0.5 µl of the overlay was mixed with 0.5 µl of 5 mg/ml α-cyano-4-hydroxycinnamic acid (CHCA) matrix solution (Laser Biolabs, Sophia-Antipolis, France) in 0.1% trifluoroacetic acid (TFA) and 50% acetonitrile. Spots were air dried and mass analysis was performed using a MALDI TOF/TOF 4700 Proteomics Analyzer (Applied Biosystems, Framingham, MA, USA). Spectra were collected in the *m/z* range of 800–5000 and peaks with a signal to noise ratio of more than 50 were selected for MS/MS acquisition. MS/MS was performed with air as collision gas at an acceleration potential of 1 kV. Protein identification was performed against the *L. lactis* MG1363 database [34], combined with human keratins (NCBI accession numbers P35908, P35527, P13645, and NP_006112), using GPS Explorer 3.5 (Applied Biosystems) and Mascot (version 1.9, Matrix Science, London, UK) as search algorithm. The MS/MS based peptide and protein identifications were further validated with the program Scaffold (version Scaffold_2_04_00, Proteome Software Inc., Portland, OR). Proteins were considered identified when at least two peptides were matched with >99.5% MS/MS confidence.

Tryptic digests of gel plugs that did not lead to identification of protein(s) using the above mentioned procedure were separated on a reverse phase ZORBAX 300SB-C18 (Agilent, Wilmington, DE USA) column on a multidimensional liquid chromatography system (Ettan MDLC, GE Healthcare, Uppsala, Sweden). Peptides were separated with a 25 min linear gradient of acetonitrile 10–80% (v/v) in water containing 0.1% TFA (v/v). Column effluent was mixed 1∶1 with 2 mg/ml CHCA matrix solution in 60% acetonitrile and 0.1% TFA with 2 nM angiotensin II (MW 1045.53 Da) and 4 nM ACTH (MW 2464.19 Da) (Sigma, Zwijndrecht, The Netherlands) as standards for internal mass calibration. Fractions of 20 s width were spotted on a blank MALDI target with the Probot system (LC Packings, Amsterdam, The Netherlands). Mass spectrometric analysis of spots and protein identification were done as described above.

### Miscellaneous assays

#### Glycolysis

The maximal glycolytic activity of GM17- and GCDM-grown *L. lactis* NZ900 cells was measured as described [35]. Cells, grown in the respective media, were harvested at OD_600_≈0.5 and washed with 0.5 mM KPi, pH 6.4, 70 mM KCl plus 1 mM MgSO_4_. The cells were then adjusted to 0.3 mg protein/ml and supplemented with glucose to a final concentration of 5 mM; fermentation rates were estimated from the recording of pH changes. The pH was monitored with a Sartorius PP-25 pH meter and the changes in pH were converted into nanomoles of H^+^ by calibration with 1 µl aliquots of 50 mM HCl. The maximal glycolytic rate was determined in the presence of the protonophore SF6847 (10 µM, final concentration).

#### Membrane potential

The energy status of the cells was estimated from the fluorescence of 3′,3′-dipropylthiadicarbocyanine [DiSC3(5)], which is a reporter of the membrane potential [36]. Preparation of the cells was done as described under glycolysis except that the cells were resuspended in 50 mM KPi, pH 6.4, to a protein concentration of 0.1 mg/ml. Membrane potential formation was monitored upon addition of glucose to a final concentration of 5 mM. The electrochemical proton gradient was dissipated by the addition of SF6847 to a final concentration of 10 µM. Fluorescence was measured with Spex Fluorolog 322 fluorescence spectrophotometer (Horiba Jobin Yvon, Park Avenue, NJ, USA). The fluorescence was measured at an emission wavelength of 666 nm with an excitation wavelength of 643 nm (both with a 5-nm band pass).

#### Fatty acids

Product formation of GM17- and GCDM-grown cells was assayed on culture supernatants. The volatile fatty acids, present in 1 ml of culture supernatant supplemented with 50 mM 3-methylbutanoic acid (Merck, Whitehouse Station, NJ, USA) as an internal standard, were extracted with 1 ml of diethyl ether after acidification of the medium with 0.1 ml of concentrated formic acid. Non volatile fatty acids (*e.g.* lactate), present in 1 ml of culture supernatant supplemented with 50 mM malonic acid (Merck, Whitehouse Station, NJ, USA), were extracted by overnight incubation with 0.5 ml of 50% H_2_SO_4_ and 1 ml of methanol. Lactate was extracted into organic solvent by the addition of 0.5 ml deionized water and 0.5 ml of methanol. The extracted fatty acids were analyzed by HP-5 (30 m long with outer diameter of 0.25 mm and inner diameter of 0.25 µm) gas chromatography (Agilent, Wilmington, DE USA). The injector and detector were maintained at 250°C while helium was used as carrier gas at a rate of 35 cm/sec. Samples were analyzed by loading 2 µl of the sample onto the column and fatty acids were separated with a linear temperature gradient from 30°C to 300°C in 25 min. The resulting peaks were quantified with a flame ionization detector (Agilent, Wilmington, DE USA).

## Supporting Information

Figure S1 Protein overexpression in GM17- and GCDM-grown *L. lactis* NZ9000. Duplicate dataset showing the reproducibility of the expression/immunoblotting experiments. For further details, see legend to Figure 1.
(0.87 MB TIF)

Figure S2 Time-resolved protein expression in GM17- and GCDM-grown cells. (Panels A and C) Growth of *L. lactis* NZ9000 in GM17 (closed symbols) and GCDM (open symbols), following the addition of 0.2% (panel A and B) or 0.05% (panel C and D) of nisin A-containing NZ9700 supernatant to a culture at OD_600_≈0.5. The cells express either OpuAC-GFP (circles) or BcaP-GFP (inverted triangles). Growth of the cells was monitored by measuring the optical density at 600 nm. (Panels B and D) Time dependence of OpuAC-GFP and BcaP-GFP expression (symbols the same as in panels A and C). Expression levels were quantitated using the GFP fluorescence of whole cells. Data were corrected for cell density.
(1.52 MB TIF)

Figure S3 Glycolytic activity and membrane potential of GCDM and GM17-grown cells of *L. lactis* NZ900. The data for GM17- and GCDM-grown cells are indicated by black and red lines, respectively. (A) Glycolytic activity: Cells at a protein concentration of 0.3 mg/ml in 0.5 mM KPi, pH 6.4, 70 mM KCl plus 1 mM MgSO4 were incubated at 30°C. At time 1 min, glucose was added to a final concentration of 5 mM, which resulted in an acidification of the medium. Glycolyzing cells convert nearly all their glucose into lactic acid (confirmed by the fatty acid analysis), i.e., 2 lactate anions plus 2 protons per glucose. The rate of acidification is thus a direct measure of the glycolytic activity. (B) Membrane potential: Cells at a protein concentration of 0.3 mg/ml in 50 mM potassium phosphate, pH 6.4, plus 3 µM DiSC3(5) were incubated at 30°C. At time 200 sec, glucose was added to a final concentration of 5 mM, which resulted in the generation of a membrane potential (observed as a decrease in fluorescence). After 400 sec, SF6847 (10 (lower case mu}M, final concentration) was added to dissipate the electrochemical proton gradient across the cell membrane.
(0.54 MB TIF)

Figure S4 Peptides with BCAAs are sufficient enough to increase recombinant protein production in GCDM grown cells. Duplicate dataset showing the reproducibility of the expression/immunoblotting experiments. For further details, see legend to Figure 5A.
(0.37 MB TIF)

Table S1 Complete list of identified proteins from 2D gels.
(0.10 MB XLS)

Table S2 Oligonucleotides used to construct the pIL-based bcaP expression vectors.
(0.03 MB DOC)
