# Supplementary figures and images for: Amino Acid Accumulation Limits the Overexpression of Proteins in Lactococcus lactis
Source: PLoS One. 2010 Apr 26;5(4):e10317. doi: 10.1371/journal.pone.0010317 (PMC2859938; doi:10.1371/journal.pone.0010317)

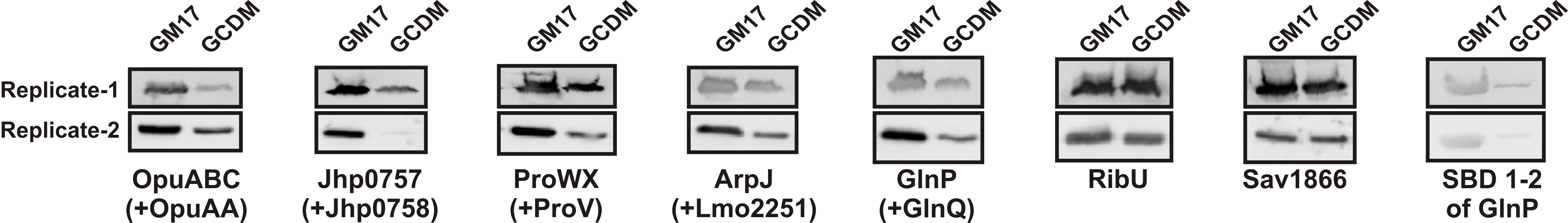

Supplement: Figure S1 — Protein overexpression in GM17- and GCDM-grown L. lactis NZ9000. Duplicate dataset showing the reproducibility of the expression/immunoblotting experiments. For further details, see legend to Figure 1. (0.87 MB TIF) [file pone.0010317.s001.tif]

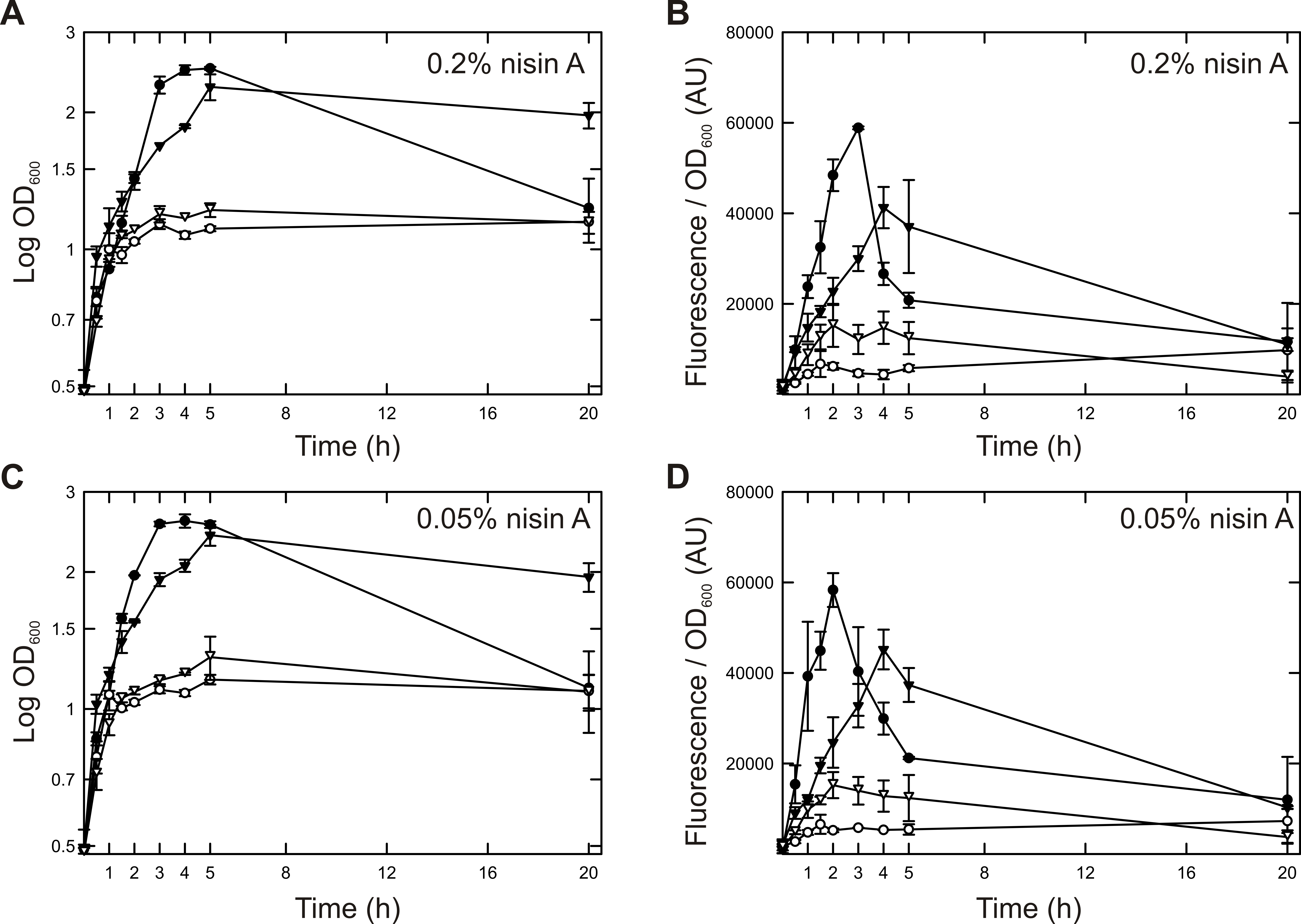

Supplement: Figure S2 — Time-resolved protein expression in GM17- and GCDM-grown cells. (Panels A and C) Growth of L. lactis NZ9000 in GM17 (closed symbols) and GCDM (open symbols), following the addition of 0.2% (panel A and B) or 0.05% (panel C and D) of nisin A-containing NZ9700 supernatant to a culture at OD600≈0.5. The cells express either OpuAC-GFP (circles) or BcaP-GFP (inverted triangles). Growth of the cells was monitored by measuring the optical density at 600 nm. (Panels B and D) Time dependence of OpuAC-GFP and BcaP-GFP expression (symbols the same as in panels A and C). Expression levels were quantitated using the GFP fluorescence of whole cells. Data were corrected for cell density. (1.52 MB TIF) [file pone.0010317.s002.tif]

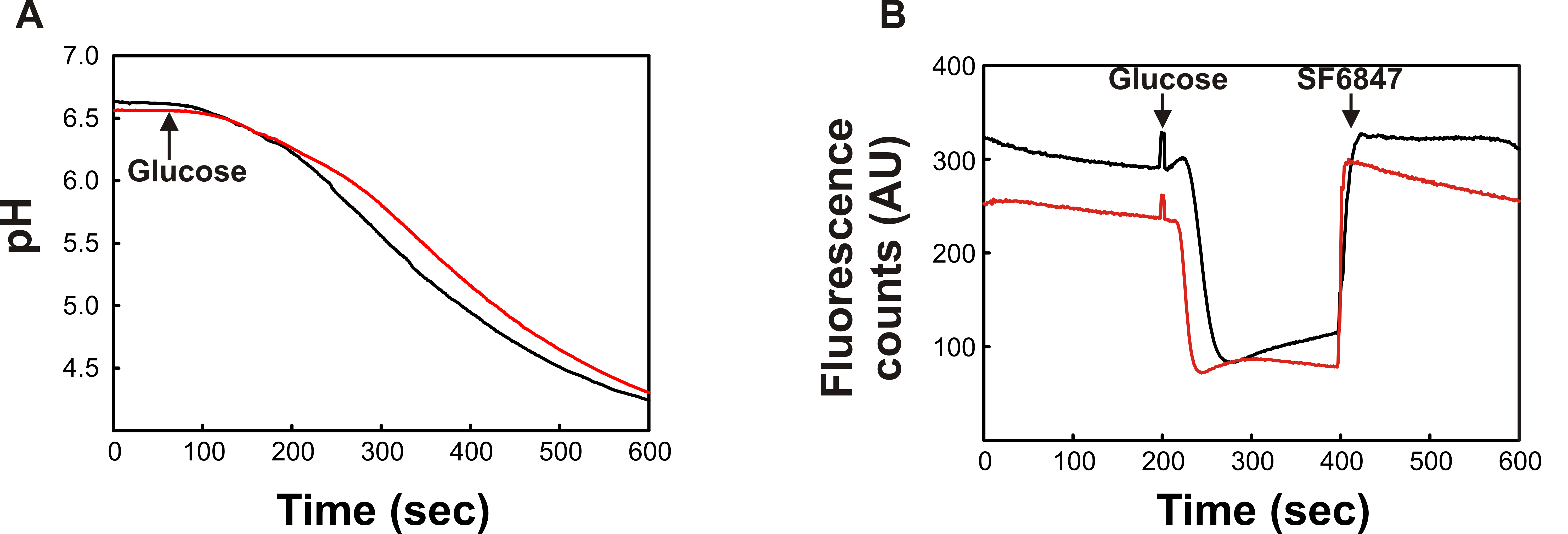

Supplement: Figure S3 — Glycolytic activity and membrane potential of GCDM and GM17-grown cells of L. lactis NZ900. The data for GM17- and GCDM-grown cells are indicated by black and red lines, respectively. (A) Glycolytic activity: Cells at a protein concentration of 0.3 mg/ml in 0.5 mM KPi, pH 6.4, 70 mM KCl plus 1 mM MgSO4 were incubated at 30°C. At time 1 min, glucose was added to a final concentration of 5 mM, which resulted in an acidification of the medium. Glycolyzing cells convert nearly all their glucose into lactic acid (confirmed by the fatty acid analysis), i.e., 2 lactate anions plus 2 protons per glucose. The rate of acidification is thus a direct measure of the glycolytic activity. (B) Membrane potential: Cells at a protein concentration of 0.3 mg/ml in 50 mM potassium phosphate, pH 6.4, plus 3 µM DiSC3(5) were incubated at 30°C. At time 200 sec, glucose was added to a final concentration of 5 mM, which resulted in the generation of a membrane potential (observed as a decrease in fluorescence). After 400 sec, SF6847 (10 (lower case mu}M, final concentration) was added to dissipate the electrochemical proton gradient across the cell membrane. (0.54 MB TIF) [file pone.0010317.s003.tif]

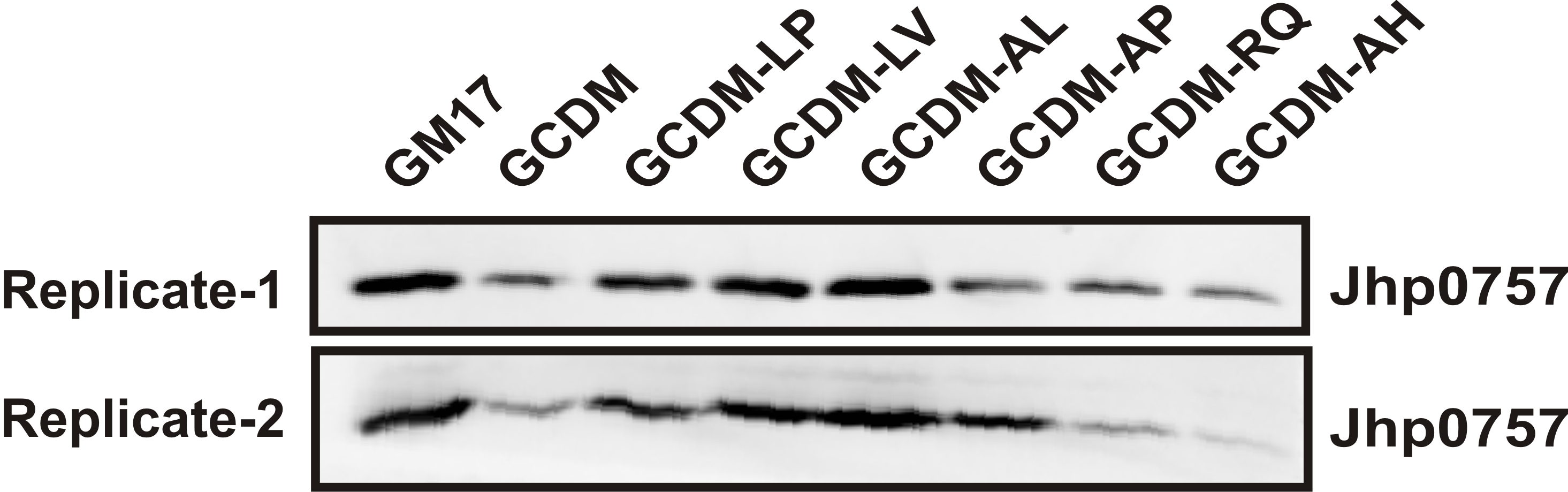

Supplement: Figure S4 — Peptides with BCAAs are sufficient enough to increase recombinant protein production in GCDM grown cells. Duplicate dataset showing the reproducibility of the expression/immunoblotting experiments. For further details, see legend to Figure 5A. (0.37 MB TIF) [file pone.0010317.s004.tif]
