# Supplementary material for: Amino Acid Accumulation Limits the Overexpression of Proteins in Lactococcus lactis
Source: PLoS One. 2010 Apr 26;5(4):e10317. doi: 10.1371/journal.pone.0010317 (PMC2859938; doi:10.1371/journal.pone.0010317)
Supplement: Table S2 — Oligonucleotides used to construct the pIL-based bcaP expression vectors. (0.03 MB DOC) [file pone.0010317.s006.doc]

**Table S2.** Oligonucleotides used to construct the pIL-based *bcaP* expression vectors.

| **Primers** | **Sequence** |
| --- | --- |
| P_32_-*Bgl*II Fwd | 5' GTCACAGATCTATGCAGCTCGAGGGGATATGATAAGATTAATAG |
| P_32_-*Nco*I Rev | 5' TTGATTAGAACCCATGGCAAAATTCCTCC |
| P_32_-*Xho*I Fwd | 5' ATGCAGCTCGAGGGGATATGATAAGATTAATAGTTTTAG |
| *bcaP-GFP* Rev | 5' CGTCACGAATTCTTAACTAGTTTTGTAGAGCTCATCCATGCC |
| *bcaP-GFP*His Rev | 5’ ATGCACGAATTCTTAATGATGGTGATGATGATGG |
